# Working memory improves developmentally as neural processes stabilize

**DOI:** 10.1371/journal.pone.0213010

**Published:** 2019-03-07

**Authors:** David Florentino Montez, Finnegan J. Calabro, Beatriz Luna

**Affiliations:** 1 Department of Neurology, Washington University, Saint Louis, Missouri, United States of America; 2 Department of Psychiatry, University of Pittsburgh, Pittsburgh, Pennsylvania, United States of America; 3 Center for the Neural Basis of Cognition, Pittsburgh, Pennsylvania, United States of America; 4 Department of Bioengineering, University of Pittsburgh, Pittsburgh, Pennsylvania, United States of America; Embedded systems for health, SWEDEN

## Abstract

Working memory performance is a key indicator of cognitive and developmental status. While recent evidence indicates that stabilizing neural gain supports the stabilization of working memory during adolescence, the computational mechanisms linking neural stabilization to behavior are poorly understood. We develop a mechanistic account of behavior during the memory-guided saccade task based on a stochastic accumulator framework. Results indicate that a specific balance of independent gain signals affecting working memory representations and oculomotor response thresholds can account for a peculiar U-shaped feature of the speed-accuracy relationship. Additionally, aspects of behavioral variability and mean behavioral performance, as well as subtle shifts in the shape of the speed-accuracy relationship across development, can be accounted for by the stabilization of these two sources of variability. Thus, the stabilization of neural variability can, in part, account for developmental improvements in behavioral variability as well as some improvement in mean behavioral performance.

## Introduction

Adolescent cognitive development is marked by stabilizing behavioral responses which occur alongside improvements in mean behavioral performance. This is seen, for instance, in the tendency for developmental trajectories of decreasing average reaction times and increasing accuracy to occur in tandem with commensurate decreases in the trial-to-trial variability of those measures [1–4]. These developmental changes are especially apparent in tasks that place demands upon cognitive processes, such as working memory (WM), and as such, metrics of mean behavioral performance and trial-to-trial behavioral variability are both often used as barometers of cognitive functioning and are widely employed as surrogate measures of developmental status or cognitive impairment [5–7].

As a marker of developmental status, behavior during oculomotor tasks is particularly sensitive and has the benefit of a long history of use in studies aiming to characterize the development of reflexive behavior and cognitive processes such as memory, attention, inhibitory control, and incentive processing. Across a variety of oculomotor task designs, researchers have observed steady improvements in most aspects behavioral performance throughout childhood and into early adulthood [8–11].

The neural circuitry supporting oculomotor performance and its development during WM tasks are delineated well and have been characterized in both humans and non-human primates [12–16]. Thus, a robust framework exists within which to interpret results and generate hypotheses about the neural computations supporting WM and their changes throughout childhood and adolescence. This has allowed for the development of computational models that account for some features oculomotor performance, such as the distribution of reaction times and proportion of correct trials, but they are rarely deployed in service of characterizing the changes in behavior that occur across development [17–19]. In addition, most computational models of oculomotor performance have been applied to tasks in which errors in behavioral performance are modeled as binary outcomes rather than to tasks that produce behaviors whose correctness may vary in degree across trials. This is particularly important in developmental research where performance in oculomotor tasks improves steadily, with eye movements on correct trials becoming more precise and more accurate. Because steady improvements in measures of mean behavioral performance and trial-to-trial variability are so often associated with maturation, a key goal of developmental neuroscience is to understand the mechanisms producing these changes.

However, since developmental changes in mean behavioral performance are often highly correlated with changes in trial-to-trial behavioral variability, the extent to which these metrics uniquely reflect developmental changes in distinct aspects of neural processing is difficult to ascertain. Moreover, for many cognitive processes, we lack sufficiently detailed mechanistic or computational accounts of how mean performance and performance variability are driven by different aspects of neural processing. Understanding what consequences adolescent maturation has for neural computations supporting cognition requires that we develop such computational accounts.

Recently, we reported fMRI evidence that the developmental stabilization of behavior during the performance of WM tasks is, in part, the result of the stabilization of multiple widespread, or global, neural gain signals [4]. While we observed a clear relationship between trial-to-trial behavioral performance and a measure of global gain fluctuations, the computational mechanism by which variable neural gain produces variable behavior remains unclear. Our goal here is to develop a computational framework with which to develop our mechanistic understanding of the link between neural variability and the development of behavioral performance during WM tasks.

One striking aspect of our previous findings is that the trial-to-trial relationship between the reaction times and accuracies of memory-guided saccades was U-shaped, such that the poorest accuracy was observed when reaction times were both excessively long and unusually short. We proposed that a U-shaped speed-accuracy relationship could arise from an appropriate balance of independent neural gain variability affecting excitatory and inhibitory activity within circuitry supporting oculomotor and WM processes. Here we formalize a computational account of our hypothesis based on a high-dimensional drift-diffusion, or race model, framework that models a continuous range of response outcomes, and demonstrate that the U-shaped speed accuracy relationship that we observed in the memory-guided saccade task can indeed arise from the appropriate balance of two independent sources of neural gain variability. The first, which modulates the amplitude of neural activity supporting spatial WM representations, is effectively excitatory in nature, while the second, responsible for modulating the response threshold within a population of oculomotor neurons, is effectively inhibitory. Notably, we find that the simultaneous stabilization of these two sources of neural gain variability can not only account for the stabilization of behavioral performance and observed speed-accuracy relationship, but accounts for a portion of the developmental improvement observed in in mean behavioral performance as well.

## Results

### Memory-guided saccades exhibit a U-shaped speed-accuracy relationship

**Fig 1:**
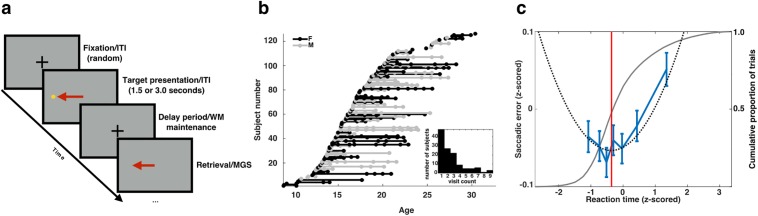
MGS task, subject cohort, and speed-accuracy relationship. a) Temporal schematic of the memory-guided saccade task employed in this study. b) The age distribution of subjects included in the mixed-longitudinal cohort. Individual sessions are depicted by dots. a-b) Reproduced from Figure 1 (Montez et al. 2017), eLife, published under the Creative Commons Attribution 4.0 International Public License (CC BY 4.0; https://creativecommons.org/licenses/by/4.0/). Return sessions performed by the same subject are connected by solid lines. Male subjects are rendered in gray, females in black. Inset histogram depicts the distribution of return sessions across the sample. c) The U-shaped relationship between reaction time and accuracy in the memory-guided saccade task. The x-axis depicts reaction times, z-scored within a session and task condition. The left-hand y-axis represents saccadic error, also rectified and normalized within session. Empirical measurements are rendered in blue. Error bars represent ±1 standard error of the mean. Behavioral data was adaptively binned so that each data point contains the same number of measurements. The smooth quadratic curve (dashed black) indicates the line of best fit for the non-binned data. The vertical red line indicates global minima of the quadratic curve, the point at which the relationship between reaction time and accuracy changes direction. The right-hand y-axis is associated with the gray curve, which depicts the cumulative distribution of trial reaction times across all sessions.

We investigated developmental change in behavioral performance during a memory-guided saccade task (MGS; Fig 1a) within a large accelerated longitudinal cohort of 126 subjects between the ages of 8 and 33 years (Fig 1b). Data from this task and details of its design have been previously reported [4,12]. During each session, subjects performed 60 trials of the memory-guided saccade (MGS) task while in an fMRI scanner. Younger subjects typically have greater difficulty suppressing pre-potent orienting saccades to unpredictably presented visual stimuli. To reduce potential age-related differences in task performance that might result from developmental differences in such pre-potency processes (i.e., inhibitory control), we allowed subjects to perform an initial visually-guided “encoding” saccade to the target stimuli. A trial began with subjects maintaining fixation on a central stimulus cross. At the start of an initial presentation interval (PI) the fixation cross was extinguished and a target appeared for one of two durations (1.5 or 3.0s) at one of six pseudo-randomly selected locations placed along the horizontal visual meridian (±3°, ±6°, and ±9°). Subjects were instructed to quickly and accurately perform a saccade toward and fixate upon the target stimulus. At the close of the PI, the target disappeared, and subjects reoriented to the central fixation cross, marking the beginning of the delay interval (DI). During the DI, subjects maintained fixation for either 1.5s or 9.0s. At the end of the DI the central fixation cross was extinguished, and subjects performed a voluntary saccade to the remembered location of the target in the absence of sensory cues. Throughout the task, subjects’ eye positions were monitored and recorded by an infrared eye-tracking system equipped with long-range optics (Model R-LRO6, Applied Science Laboratory). For each trial we measured reaction time (RT), the duration separating the end of the DI and the initiation of the MGS, and saccadic error (SE), the horizontal angular distance between the location of the target and the end point of the MGS.

The developmental changes in measures of mean behavioral performance and overall behavioral variability in this task have been reported previously [4]. Here our interest was in understanding trial-to-trial behavioral variability; thus, we examined the relationship between RT and SE of responses at the single trial level. We computed RT values, z-scored separately within each of the four task conditions. To account for session-to-session differences in calibration of the eye-tracker, we z-scored SE within each task condition and then rectified those values by discarding the sign of the error. This transformation implicitly combines errors produced by saccades that overshoot and undershoot their mark, reducing them to a value that conveys simply how far from the target the saccade has landed. The resulting SE values were then z-scored a second time allowing for negative values. This transformation simply re-centers and scales the data and allows small and large SE to be distinguished readily by their sign. In employing this procedure, we ignored inter-subject, inter-session, and between task condition differences in performance, and limited ourselves to measuring normalized trial-to-trial behavioral variability.

RT and SE exhibit a U-shaped relationship in which there appears to be two distinct regimes of speed-accuracy correlation (Fig 1c). For trials with the fastest RTs, decreasing RT (slowing down) is associated with decreases in SE (improved accuracy), as with traditional speed-accuracy relationships. However, for trials with the slowest RTs, this relationship is reversed. We determined whether both portions of the speed-accuracy relationship were comprised of a similar proportion of trials by estimating the best fitting quadratic curve and calculating its global minima, the point at which the direction of the speed-accuracy relationship reverses. Comparing the location of the global minima against the empirical cumulative distribution of RTs, we determined that roughly 52% of trials exhibited traditional speed-accuracy trade-off characteristics. Our regression analyses support this observation, showing that SE has a significant quadratic relationship with RT (t(16362) = 3.97; p = 7.31e-5), but no significant linear effect (t(16362) = 1.4;p = 0.16). In our analyses, we accounted for systematic differences in RT and SE arising from the spatial location of the targets by including each of the six possible target locations as a categorical nuisance regressor. There were no significant main effects of, or significant interactions with, subject age in the quadratic model (all p > 0.1). However, in a model in which age interactions are limited to only the linear reaction time term (no Age*RT^2^ term), we noted a trend-level significance (p = 0.07). Thus, when assessed with a traditional quadratic linear model, age-related changes in the U-shaped speed-accuracy relationship are minimal.

### A mechanistic model of memory-guided saccade performance

**Fig 2:**
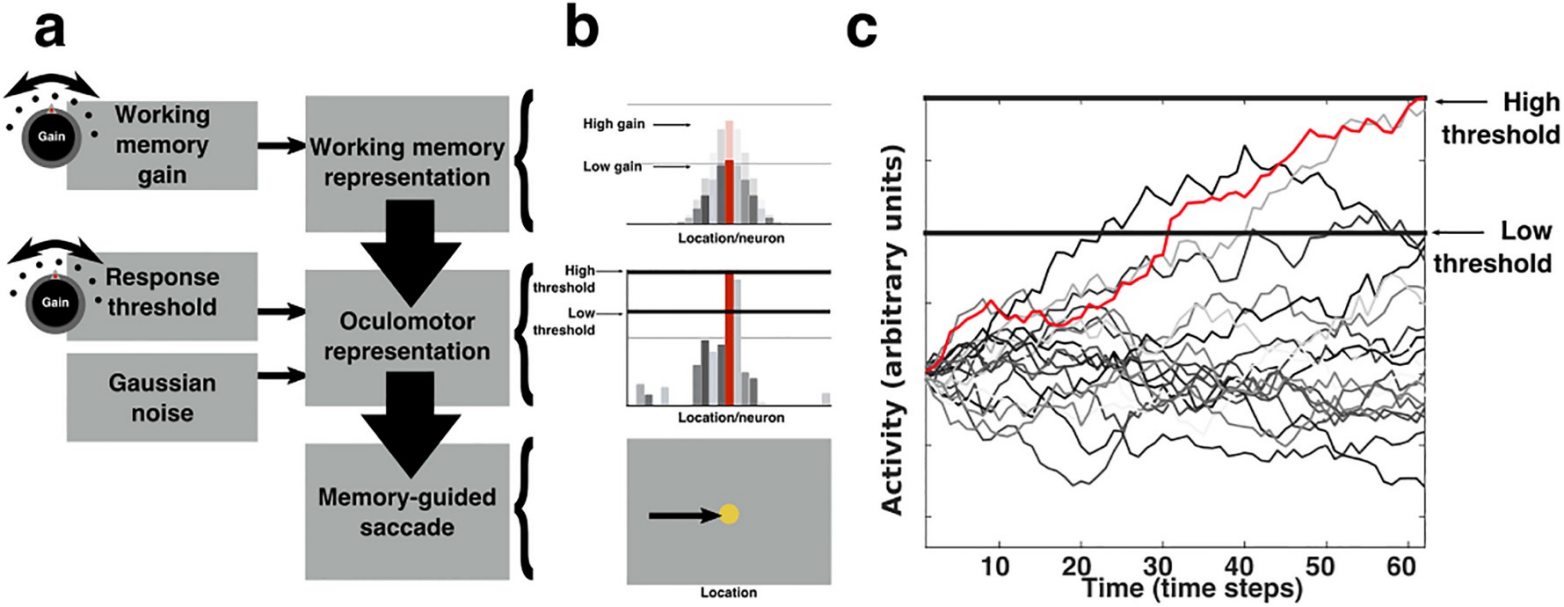
A mechanistic model of MGS performance. a) MGS performance is modeled as the transfer over time of a topographic WM representation into a topographic representation of an oculomotor response. In addition to accumulating Gaussian random noise, key sources of neural variability include two independent gain modulating signals, one that affects the amplitude of WM activity, and a second that modulates the response threshold of the oculomotor population. b) Depicts the state of simulated neural activity within WM and oculomotor populations at an arbitrary time point during a trial. The activity of each neuron codes either for the location of a remembered target or a saccade. The height of each bar represents the activity of a single neuron. The neuron representing the location closest to the remembered target is rendered in red. Gain variability affects WM by multiplicatively scaling the population activity (top). The effect of gain variability on the oculomotor population is the modulation of the response threshold (middle). The peak of activity within WM and oculomotor populations coincides with the remembered location of the stimulus and subsequent memory-guided saccade (bottom). c) An example time course of accumulating activity within the oculomotor population for a trial. Each line corresponds to the state of a particular oculomotor neuron across time; coloring as in b. A simulated memory-guided saccade is performed once any neuron in the oculomotor population passes the response threshold.

Memory-guided saccades are performed in the absence of stimulus-relevant visual input and are guided solely by a representation of the target maintained in WM. Because the neurons most proximally related to the production of a saccade are distinct from those neurons supporting WM representations, producing a memory-guided saccade, at a minimum, is likely to involve a process whereby WM’s spatial information is transferred, or “read out”, into a second pattern of oculomotor-related neurons whose activity directly contributes to the production of a corresponding saccadic eye-movement (Fig 2a) [13,20]. We modeled this read out process using a simple extension of the stochastic accumulator framework (S1 Text), which has been used previously to account for behavioral responses and speed-accuracy relationships in other contexts [21–24].

Specifically, we modeled two separate populations of neurons, corresponding to WM and oculomotor circuitry, as vector representations of the one-dimensional visual field (i.e., along the horizontal meridian) in which each element of a vector corresponds to the activity of a simulated neuron. In both cases, WM and oculomotor vectors are organized so that each of their elements corresponds to a neuron whose activity represents a particular spatial location, and are so ordered that adjacent entries correspond to neurons representing adjacent spatial locations. In this model, a remembered stimulus is encoded by heightened activity amongst the set of WM neurons representing the locations most near the target (Fig 2b). This heightened activity has a unit Gaussian profile with a peak centered on the WM neuron corresponding to the remembered target location.

The read out of WM information into the oculomotor representation begins with the extinction of the fixation stimulus, which cues the subject to perform a memory-guided saccade. For the sake of simplicity, we omit from our mode potential neural variability that may arise during the sensory and cognitive processing that accompanies this cue and take as our starting point the initial transformation of a WM representation into an appropriate saccadic response. We model this process as the noisy accumulation over time of the WM representation into the oculomotor representation. That is, at each time step of a simulated trial, a scalar multiple of the state of the WM population is added to the state of the oculomotor population along with Gaussian random noise that affects each oculomotor neuron independently. This noisy accumulation process continues until such time as any neuron within the oculomotor population achieves a value that surpasses a response threshold, at which point a simulated saccade is performed to the location associated with the oculomotor neuron that crosses the threshold first (Fig 2c). This construction allows for the modeling of continuous reaction time distributions as well as continuous error distributions, similar to how we have measured performance on the MGS task.

Three sources of neural variability affect the transfer of information between WM and oculomotor representations in this model (Fig 2a and 2b). The first source of neural variability, mentioned above, is independent Gaussian random noise that accumulates over time within the activity of the simulated oculomotor neurons. It is the presence of this source of neural variability that allows for the possibility of response errors of varying magnitude; in its absence, there can be no variability in the endpoints of simulated saccades. As in all stochastic accumulation models, it also provides a key source of trial-to-trial reaction time variability. The second source of neural variability is trial-to-trial fluctuations in gain that affects the amplitude of neural activity comprising the WM representation. WM gain variability is modeled as trial-to-trial differences in the amplitude of the pattern of activity present within the simulated population of WM neurons (Fig 2b; top). Because a scalar multiple of the current WM state is added to the oculomotor state at each time step, changes in WM gain affect the rate at which the WM representation accumulates, or is read out, into the oculomotor representation. All other things being equal, increases in WM gain will decrease simulated RT because fewer time steps are required in order for the oculomotor neurons to surpass the response threshold. The third source of variability is trial-to-trial fluctuations in the height of the oculomotor response threshold. Differences in the oculomotor response threshold alter the magnitude of activity required in order to evoke a saccade (Fig 2b; middle). In the absence of other changes, increasing the height of the oculomotor response threshold will tend to increase simulated RT, as it will take a greater number of time steps for the neurons within the oculomotor population to surpass this greater threshold value. In the present implementation, all sources of stochastic variability are statistically independent. We note that this modeling framework is amenable to numerical analysis, and exact expressions for reaction time and saccadic error distributions can be calculated in lieu of performing many simulations and then approximating response distributions (see Material and methods). Here we report results based on calculated, rather than simulated responses (although see Supporting information for validation).

### A balance of independent gain variability affecting working memory and oculomotor response threshold accounts for the U-shaped speed-accuracy curve

We examined how trial-to-trial variability in gain signals that affect the amplitude of WM representations and the height of oculomotor response thresholds shape the speed-accuracy relationship during the memory-guided saccade task. Previous literature indicates that trial-to-trial variability affecting the response threshold in stochastic accumulator models can account for the presence of a traditional speed-accuracy relationship in which faster responses are associated with greater error [22,25]. In other contexts, researchers have noted that trial-to-trial variability affecting the drift rate in accumulator models—here akin to WM gain variability—can produce a contrary effect in which error trials tend to have longer reaction times [21,23]. Given the opposing effects that response threshold and WM gain variability could have on the structure of the speed accuracy relationship, we explored whether an appropriate balance between the two might be struck so as to account for the U-shaped speed-accuracy profile that we observed in our data.

**Fig 3:**
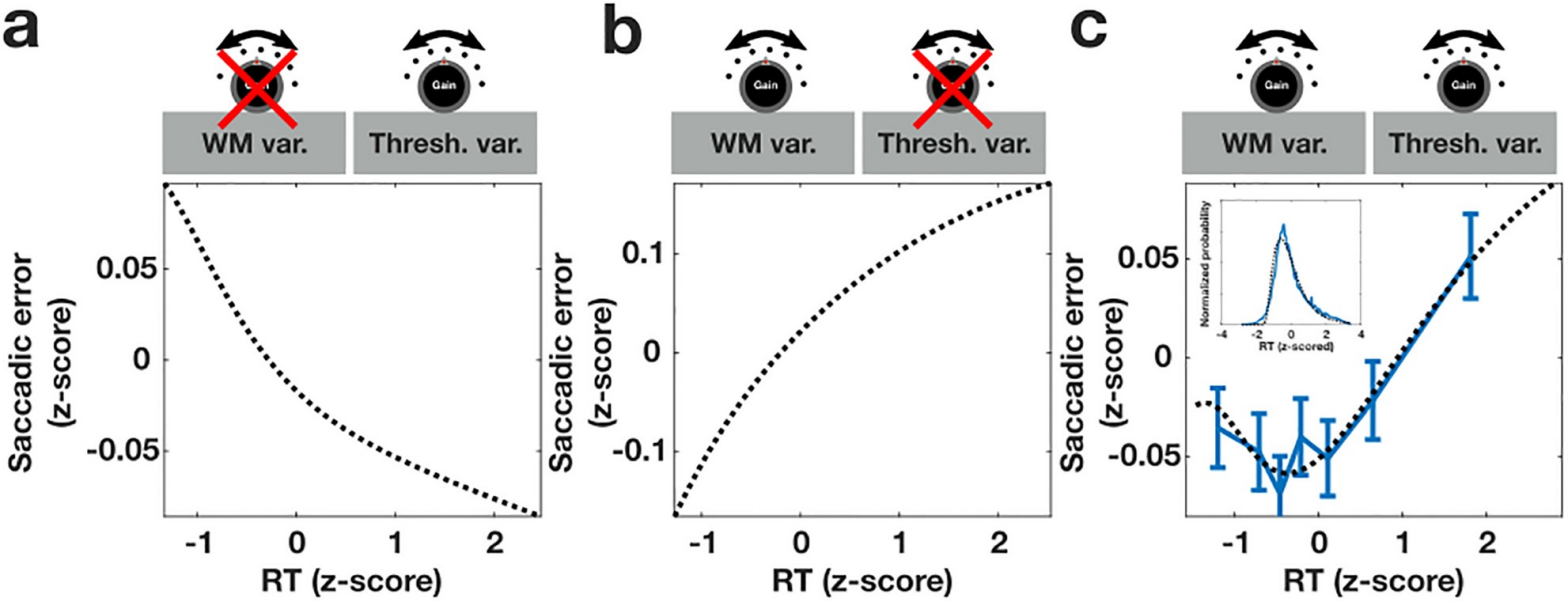
The effect of sources of gain variability on the speed-accuracy relationship. a) The speed-accuracy curve produced when including trial-to-trial oculomotor response threshold variability, but not gain variability. b) The speed-accuracy curve produced when including WM gain variability, but not threshold variability. c) The speed-accuracy curve resulting from the best fitting model with balanced independent WM and oculomotor threshold variability. Inset depicts the calculated z-scored reaction time distribution compared to the empirical z-scored reaction time distribution across all sessions. Dashed black lines indicates calculated speed-accuracy curves, blue indicates empirical data. All examples include accumulating Gaussian noise variability. For all calculations the size of the WM and oculomotor populations was n = 11 neurons. Sustained WM activity representing the location of the target was modeled as a Gaussian peak of unit magnitude and variance centered on the neuron represent the target location. Neurons represented a region of visual space evenly spanning ±4 standard deviations. The mean amplitude of the WM population was equal to 2 and the mean response threshold was held constant at 200. Regarding the three free model parameters: The standard deviation of the accumulating Gaussian noise, **σ** = **12.2775**; the width of the uniform distributions of response thresholds, **τ** = **118.0400** and WM gain values, **ω** = **2.4952**. Speed-accuracy curves for a and b were computed by setting **ω** and **τ** equal to zero respectively.

First, we verified that this model could reproduce the anticipated effect of a variable response threshold in isolation. We computed the speed-accuracy relationship for an ensemble of trials in which only the oculomotor response threshold varied across trials following a uniform distribution (see Materials and methods). As expected, we observed that when WM gain variability is absent across trials, and the only sources of variability are trial-to-trial fluctuations in the oculomotor response threshold and accumulating Gaussian noise, responses exhibit a traditional speed-accuracy relationship in which longer RT trials are associated with lower SE (Fig 3a). The speed-accuracy relationship takes this form because fast RT trials tend to be those in which the response threshold is low; the reduced response threshold increases the likelihood of a random oculomotor neuron crossing the threshold just by chance and evoking an inaccurate saccade. Next, we examined the influence of WM gain (drift-rate) variability in the absence of response threshold variability. We computed the speed-accuracy curve produced by an ensemble of trials with variable WM gain, also drawn from a uniform distribution, and constant response threshold. In this case, we observed a qualitatively different relationship in which trials with the fastest RT tended to have reduced SE compared to slower RT trials (Fig 3b). This form of speed-accuracy relationship emerges because trials with reduced WM gain are those in which the signal to noise ratio of the target representation within the WM population is low–resulting in greater SE–and RTs are longer–due to an overall slower rate of accumulation toward the oculomotor response threshold.

To determine whether a balance of WM and oculomotor response threshold variability can produce a U-shaped speed-accuracy curve, we fit the normalized trial-to-trial empirical speed-accuracy data using a model in which both the degree of WM and oculomotor threshold variability and the standard deviation of the accumulating Gaussian noise were free parameters. Average threshold and WM gain values were held constant (see Materials and methods). Fig 3c demonstrates that the U-shaped speed-accuracy curve that we observed in our data is accounted for by appropriately balancing these sources of neural variability (t(16368) = 5.2; p = 2.018e-7)). We verified the outcome of our numerical calculations by implementing the equivalent drift diffusion model in simulation and constructing a speed-accuracy curve based on simulated behavioral responses from 6x10^6^ trials of the MGS task and found that it to be in close agreement with our numerical results (S1 Fig).

### Tandem stabilization of gain affecting working memory amplitude and response threshold height accounts for developmental changes in behavioral performance

**Fig 4:**
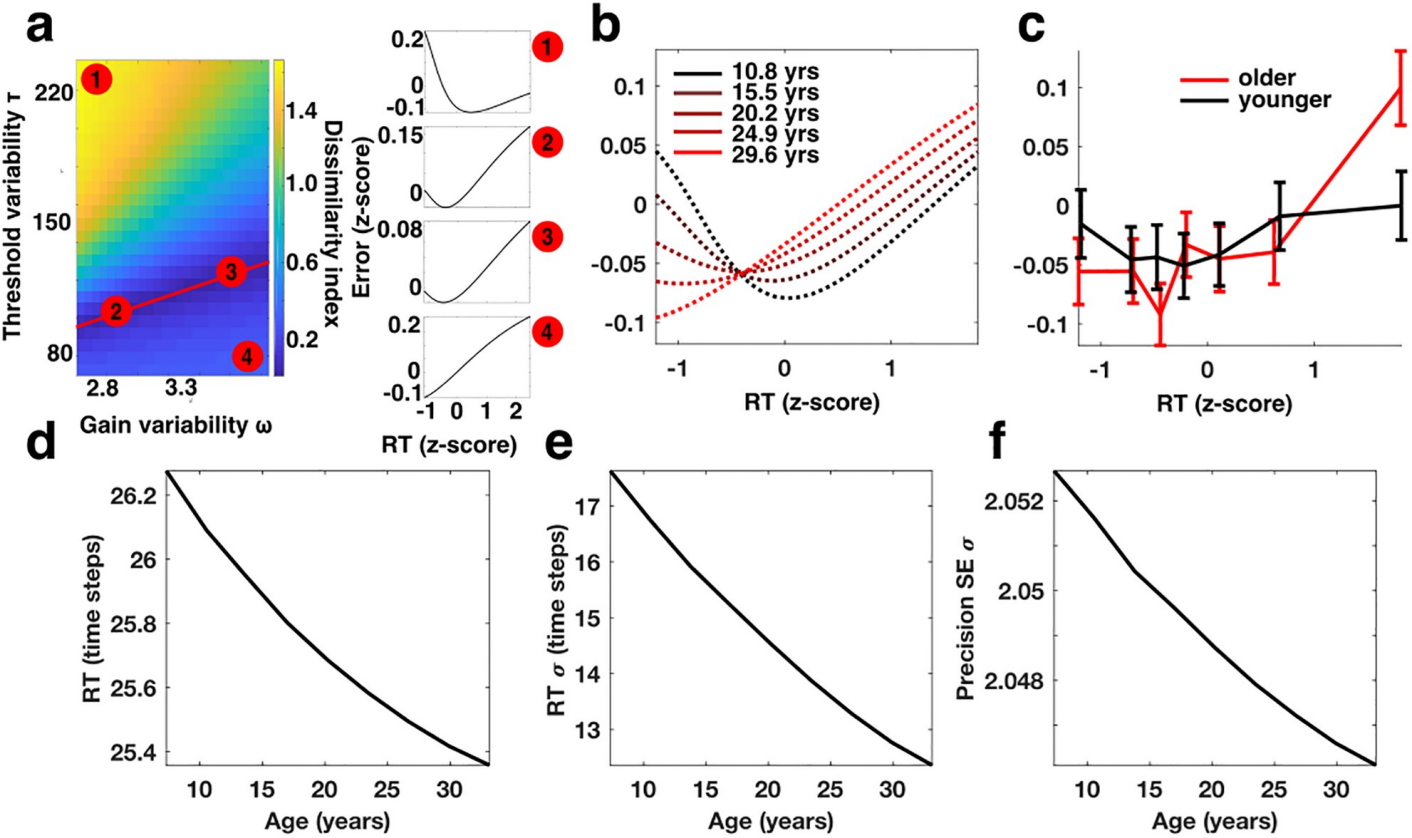
The effect of stabilizing variability during development. a) The shape of the speed-accuracy relationship changes depending on the balance of WM gain variability (x-axis) and response threshold variability (y-axis). The color depicts the dissimilarity in shape (magnitude of the vector difference) between the speed-accuracy curve determined to fit the behavioral data best (Fig 3c) and the speed-accuracy curve for other values of WM gain and response threshold variability. The red line depicts a region in parameter space in which WM gain and threshold variability produces nearly identical U-shaped speed-accuracy curves similar to that which is observed in the empirical data. Numbered side panels (1–4) are illustrative examples of speed-accuracy curves computed from different regions of the parameter space corresponding to the matched number in the heat plot. b) Best fitting age-dependent speed-accuracy curves estimated from the mechanistic model of MGS performance. c) The empirical speed-accuracy curves (as in Fig 3c) after dividing the data into younger (range: 8.8–20.0 years; mean: 16.3 years; standard deviation: 2.5 years) and older (range: 20.0–32.6 years; mean: 24 years; standard deviation: 3.03 years) age groups defined by a median split. For both b and c, the x-axis corresponds to z-scored RT and the y-axis corresponds to z-scored and rectified SE. The apparent Age*RT interaction is does not reach statistical significance when a traditional linear model is applied. However significant age-related changes in the speed-accuracy relationship are apparent when the behavioral data is fit using the mechanistic model. Expected age-related changes in d) mean RT; e) the standard deviation of RT; and f) the precision (standard deviation of SE) of MGSs estimated from the best fitting parameters of the developmental mechanistic model.

In order to characterize the role of balanced WM and oculomotor response threshold variability in shaping the behavioral responses, we compared the speed-accuracy curve produced across a range of values while holding all other parameters constant (Fig 4a). Specifically, we compared the shape of speed-accuracy curve that fit the behavioral data best (Fig 3c), and the speed-accuracy curve produced by each pair of WM gain and threshold variability values selected from the surrounding region of the parameter space. To quantify the difference in shape, we computed a dissimilarity index, defined to be the magnitude of the difference between normalized SE vectors estimated for a range of z-scored RTs (see, for instance Fig 4a side panels). We found that the local minima of the dissimilarity index demark a roughly linear region of parameter space in which a balance in WM gain and response threshold variability produces nearly identical U-shaped speed-accuracy curves (Fig 4a, red line, and side panels 2,3). Thus, the presence of a U-shaped speed-accuracy relationship within our behavioral data may suggest a particular balance, or ratio, of variability across a range of values rather than a specific fixed magnitude.

Our earlier analyses indicate that developmental changes in the profile of the U-shaped speed-accuracy curve are minimal (insignificant Age*RT and Age*RT^2^ terms). Those results, however, are obtained by fitting a non-mechanistic model that explained saccadic error as a weighted combination of linear and quadratic functions of reaction time. We therefore set out to determine whether the mechanistic model of memory-guided saccade performance provided greater sensitivity for detecting age-related changes in the speed-accuracy relationship. We refit the normalized trial-to-trial speed-accuracy data with our model, treating each visit as an independent measure, but now included two additional terms allowing the variability of gain signals affecting WM amplitude and response threshold height to vary linearly with age (see Materials and methods). The best fitting model, we found, was one in which both WM and threshold variability decrease with age. Parameter estimates from the model fit indicate that between the ages of 8.8 and 32 years, threshold variability decreases by roughly 73% while WM gain variability decreases by a more modest 3.5%. In order to understand the effects that stabilizing these sources of neural variability have on the shape of the speed-accuracy relationship, we examined the model predicted speed-accuracy curves for a range of ages (Fig 4b). These results indicate that the speed-accuracy relationship actually does change across development. Specifically, the behavioral data is consistent with a developmental trajectory in which gain variability is initially balanced so as to produce a U-shaped speed accuracy curve at younger ages. During development reductions in gain variability, predominantly affecting the oculomotor response threshold, produces a speed-accuracy relationship dominated by variability affecting WM-processes in older subjects (for instance, see Fig 3b). The result is an apparent Age*RT interaction. Fig 4c depicts the empirical speed-accuracy curves for the youngest and oldest halves of our data set defined by median split. That this interaction did not reach significance in the quadratic linear model suggests that it is suboptimal for detecting these subtle changes in the speed-accuracy relationship across development, and that the shape of these relationships is characterized better by the mechanistic model than as a strictly quadratic effect.

To quantify the significance of the developmental terms in the mechanistic model, we decomposed the full model predictions for SE into two age-related and non-age-related components. First, we computed the best fitting speed-accuracy profile for a model with no age-related terms (as in Fig 3c) and constructed an estimate of SE for each trial in our data set. This vector we defined as the non-age-related component. Then we constructed an estimate of SE using a model in which WM and response threshold variability changed linearly with age. We constructed the age-related component by residualizing the full model estimate of trial-to-trial SE with respect to the non-age-related estimate. Finally, we fit the normalized speed-accuracy data with a linear model that included both age- and non-age-related terms as separate regressors along with a constant term. We found that both age- and non-age-related terms were significant predictors of the speed-accuracy relationship (t(16367) = 4.0674; p = 4.7762e-05) and (t(16367) = 3.1586; p = 0.0016) respectively. We compared the performance of the model that included both age-related and non-age-related terms to a model containing only the non-age-related term using a simulated likelihood ratio test with 5000 iterations and found that AIC was significantly lower for the model containing the additional age-related term (non-age-related model: DOF = 3; AIC = 44889; age-related model: DOF = 4; AIC = 44881; p = 0.0014).

Finally, we explored the effect of developmental changes in WM and oculomotor threshold variability on overall measures of mean behavioral performance and behavioral variability. Using the best fitting model that included age-related changes in gain and threshold variability, we computed estimates for the mean and standard deviation of RT as well as saccadic precision across a range of age between 8 and 32 years (Fig 4c and 4d). We observed that the effect of stabilizing WM and oculomotor threshold variability is expected to produce improvements in each of these measures. In particular, we found that the developmental stabilization of WM and oculomotor threshold variability produces roughly a 3.1% reduction in mean RT (Fig 4c); a 28% reduction in the standard deviation of RT (Fig 4d); and a modest 0.29% improvement in precision (Fig 4e). Previously published analyses of this behavioral data set have shown that during the age range in question mean RT improves by approximately 39%; the standard deviation of RT reduces by 67%; and the standard deviation of MGS endpoints (imprecision) reduces by 35%. Our modeling results therefore suggest that about 8% (3.1/39) of the total developmental change in mean RT can be attributed to the stabilization of variability and also accounts for 41.8% (28/67) and a mere 0.83% (0.29/35) of the developmental improvements in the standard deviations of RT and SE (imprecision) respectively. Thus, in addition, to accounting for developmental changes in the shape of the speed-accuracy relationship, the stabilization of working and oculomotor threshold variability can account for a portion of the improvements in overall measures of mean behavioral performance and behavioral variability.

## Discussion

Average behavioral performance and behavioral variability during working memory (WM) tasks reflect important aspects of cognitive and neurological functioning, particularly during childhood and adolescent development. Understanding the computational principles underlying WM processes and their influence on behavior is key to understanding the nature of the changes that occur in the brain during normative development and during the emergence of many psychiatric disorders.

We found that a simple computational model based on a high-dimensional extension of the stochastic accumulator, or drift diffusion, framework provides an effective model for trial-to-trial variability in the memory-guided saccade task. In this model, the processes involved in producing a memory-guided saccade are approximated as the noisy diffusion of a topographic WM representation into a similarly topographic oculomotor representation, which, upon reaching a threshold value, evokes a saccade. Our results indicate that a particular balance of two functionally distinct and temporally uncorrelated sources of neural gain variability can account for the U-shaped speed accuracy relationship that we observed in the memory-guided saccade task. One component of this variability, oculomotor response threshold variability, is effectively suppressive, its primary effect being to increase the input threshold of oculomotor neurons whose activity is directly involved in the execution of a saccadic eye movement. The second source of variability, WM gain variability, is effectively excitatory, increasing the effectiveness of WM inputs projecting onto the oculomotor system. In isolation, each of these types of neural variability produce a different form of speed-accuracy relationship; in the case of oculomotor threshold variability, slower trials tend to be more accurate and faster trials less so. WM gain variability tends to have an opposing effect in which the fastest trials are most accurate, and the slowest trials are the least accurate. Balanced appropriately, these two kinds of variability produce a U-shaped speed-accuracy curve that is observed empirically. Thus, our model is consistent with recent findings in humans and non-human primates which indicates that trial-to-trial fluctuations in behavioral performance are, in part, due to the variability affecting multiple independent ongoing neural gain signals [4,26].

Our findings provide important insight into the nature of adolescent behavioral variability as well. In particular, we found that subtle developmental changes in the shape of the speed-accuracy relationship were accounted for by a reduction in both WM gain variability and oculomotor threshold variability. Different developmental rates of stabilization of WM gain and oculomotor threshold variability affects the balance of neural variability, producing developmental shifts in the shape of the speed-accuracy relationship; While our modeling results indicate that overall neural variability is lower in adults, WM gain variability contributes disproportionately. Adolescent development therefore is likely supported by the stabilization multiple sources of neural variability at different rates. The relatively poor performance of a traditional general linear model compared to the mechanistic model in detecting age-related changes in the speed-accuracy trade-off, suggest that the mechanistic model is a more appropriate model of behavioral performance. As a consequence of this sensitivity and the mechanistic interpretability of the model parameters (neural gain variability), this model may provide some utility as a diagnostic or exploratory tool for researchers studying psychiatric or neurological conditions that may affect neural gain variability.

We also observed that the trajectories of stabilizing WM gain and oculomotor response threshold variability produce curvilinear changes in average reaction time, reaction time variability, and saccadic error, which are qualitatively similar to actual developmental data. It is important to note that the model predictions of developmental changes in mean behavioral performance and overall behavioral variability are based on fitting the mechanistic model of the speed-accuracy relationship to normalized (z-scored) trial-to-trial behavioral variability within each session, which does not contain any information about mean behavioral performance and the overall magnitude of behavioral variability. Thus, developmental improvements in mean behavior and overall behavioral variability are, to some extent, emergent properties of the same developmental mechanisms that produce the shift in the shape of the speed-accuracy relationship during maturation. We therefore propose that the stabilization of WM gain and oculomotor response thresholds may be a key factor in the developmental improvements in both mean behavior and behavioral variability.

Although our model system is agnostic regarding the exact anatomy in which it is instantiated, results from prior research offer suggestions about how it may be implemented in the brain. WM representations appear to be widely distributed across the cortex, mainly within the regions that represent the corresponding sensory modalities that are being remembered [27–29]. Thus, the WM representation in our model is likely to have many contributing support regions. The superior colliculus, containing neurons whose activity represents a retinotopic map of saccadic trajectories, is plausible site for the instantiation of the oculomotor representation of our model [30]. Reductions in GABAergic input from the substantia nigra to the superior colliculus result in changes in saccade metrics that are particularly pronounced for memory-guided saccades and consistent with the lowering of a response threshold [31]. The role of GABA in setting a response threshold is also strongly suggested by simulation studies of cortico-striatal-collicular interaction [32]. We note also, that while this model treats WM and oculomotor neurons as effectively separate functional populations, it by no means implies that they must reside in separate regions of the brain. For instance, both parietal and prefrontal regions contain distinct intermixed populations of neurons whose activity appears to code for various combinations of cue, delay, and saccadic response during the performance of the memory-guided saccade task [33].

A variety of mechanisms may contribute to modulation of gain signals affecting WM. For instance, cortical gain is affected by many cognitive and biophysical processes. Norepinephrine [34], acetylcholine [35], and dopamine [36] are known gain modulators implicated in arousal and the allocation of spatial attention; indeed, simply changing the levels of background synaptic input can alter neuronal gain [37]. These critical neurotransmitter systems may be undergoing important specialization through adolescence as adult level function is being established resulting in greater stability through development.

The simple mechanistic model of memory-guided saccade performance that we have presented considers only three potential sources of neural variability: 1) the stochastic accumulation of input into a population of neurons responsible for producing an eye-movement; 2) trial-to-trial variability in gain signals that affect the amplitude of neural activity supporting WM representations; and 3) trial-to-trial variability of neural gain signals that alter the response thresholds of oculomotor neurons. The balance of these sources of variability can account for the peculiar U-shape speed-accuracy relationship that we observed. Undoubtedly though, accounting for the full scope of trial-to-trial behavioral variability will require further elaboration of the present model.

Potential avenues for elaboration include explicit modeling of trial-to-trial differences in the time taken to process the response cue and potential interactions with ongoing gain and threshold variability processes. Secondly, the temporal evolution of threshold and WM gain signals across the duration of a trial can be explicitly modelled as collapsing bound and urgency signals [38]. Additionally, given the known differences in circuitry underlying vertical and horizontal oculomotor control, a different balance of working memory gain and threshold variability may prevail when performing memory-guided saccades that have a vertical component, potentially altering the shape of the speed-accuracy curves and their developmental trajectories [39].

It is notable that the variant of the memory-guided saccade task employed in our design differed from the classical memory-guided saccade task in requiring subjects to perform an initial encoding saccade. It is conceivable that under these conditions the WM representations of remembered target locations are augmented by additional information contained in a remembered “motor trace”, information that is not available during the typical memory-guided saccade task. How this potential additional source of spatial information would alter the speed accuracy curve would vary depending on the structure of the correlated neural variability affecting the WM motor trace and the remainder of the WM population. In the case of independent variability within motor and non-motor WM representations, the read-out process could average out the noise by pooling the combined WM signals together resulting in a net reduction of WM gain variability. This would bias the speed-accuracy curve toward the shape associated with a higher proportion of response threshold variability. This hypothetical highlights the potential utility of this model in interpreting the neural computational effects of task manipulations by examining their influence on the resulting speed-accuracy curve.

These findings lend support to an emerging model of adolescent development in which the stabilization of behavior and, to some extent even improvements in mean behavioral performance, are driven by the stabilization of sources of neural variability. This suggests that developmental changes in neural variability, rather than changes in mean levels of neural activity, may play a dominant role in shaping adolescent behavior more generally.

## Materials and methods

### Subjects

We tested 152 subjects between the ages of 8 and 33. Subjects were recruited between the ages of 8 and 30 years and many returned approximately annually over the course of 1–10 years. Because this data was collected as part of an fMRI experiment, subjects were included based on a combination of behavioral and motion-related criteria: Namely, 1) Mean frame-wise displacement (FD) was less than 0.15 mm; and (2) at least 50% of the trials from each of four trial types had to be measurably correct. Incorrect trials are those for which measurements of reaction time and endpoints for both visually- and memory-guided saccades were unavailable due to blink artifacts, noisy data, or transient loss of pupil- or corneal reflection-lock. After applying these exclusion parameters our dataset consisted of 126 subjects (60 female). Participants and/or their legal guardians provided informed consent before participating in this study. Experimental procedures for this study complied with the Code of Ethics of the World Medical Association (1964; Declaration of Helsinki) and were approved by the Institutional Review Board at the University of Pittsburgh. Subjects were paid for their participation in the study.

### Fitting computational model to behavioral data

We determined the parameters resulting in the best fit of the calculated speed-accuracy data to the group average speed-accuracy data by using a direct search optimization procedure (patternsearch, MATLAB). In this procedure, we computed the expected speed-accuracy curve relating z-scored reaction time to normalized, rectified and z-scored saccadic error for a given set of parameters. We then constructed a lookup table which used to predict saccadic error from reaction time and interpolated between values using a shape-preserving piecewise cubic polynomial interpolation. Using the lookup table, we estimated saccadic error for each trial of our data set based on its z-scored reaction time. The output of the objective function was defined to be the sum-of-squared error of the difference between the empirical and estimated measures of saccadic error.

There were three free parameters in the model: 1) ***σ***, the standard deviation of the accumulating Gaussian noise; 2) ***τ***, the full width of the uniform probability distribution defining the oculomotor response threshold across trials; and 3) ***ω***, the full width of the uniform probability distribution defining working memory gain across trials. The mean value for the oculomotor response threshold was selected to be 200. The mean value of working memory gain was selected to be 2. These values were selected to provide appropriated dynamic range in possible reaction time values but are somewhat arbitrary as simply scaling gain and threshold values can results in identical models fits that differ by multiplicative factor. Developmental effects were estimated using a similar procedure with two additional parameters allowing for ***τ*** and ***ω*** to vary linearly with age.

## Supporting information

S1 Text An exact expression for the probability distributons of reactions times resulting from an accumulator race to threshold model. Here we provide a step-by-step derivation of reaction time distributions beginning with a single accumulator model and building to an ensemble accumulator model.
(DOCX)

S1 Fig Agreement between simulated and calculated results. To verify the performance of our numerical calculations, we simulated 6×10^6^ trials using a drift diffusion/race model in which parameters were selected to match those determined to fit the empirical speed accuracy data best. The yellow line depicts the best fitting 15^th^ order polynomial fit to the simulated speed-accuracy curve. The light yellow envelope represents the 95% prediction interval. The dashed line corresponds to the calculated speed-accuracy curve.
(TIFF)
