# Supplementary material for: Working memory improves developmentally as neural processes stabilize
Source: PLoS One. 2019 Mar 7;14(3):e0213010. doi: 10.1371/journal.pone.0213010 (PMC6405198; doi:10.1371/journal.pone.0213010)
Supplement: S1 Text — Here we provide a step-by-step derivation of reaction time distributions beginning with a single accumulator model and building to an ensemble accumulator model. (DOCX) [file pone.0213010.s001.docx]

A typical drift diffusion or race model simulates the evolution of a group of random variables, here referred to as accumulators, as their values increment stochastically over time. Two key features of these models are of concern: 1) the probability distribution of times required for at least one accumulator to reach some threshold value, $\boldsymbol{T}$; and 2) The probability distribution describing which accumulators tend to reach the threshold value first at a particular time. While it is straightforward to estimate these distributions by simulating many trials computationally, this approach is both time consuming and introduces a degree of noise that makes fitting simulated data to empirical data difficult and less replicable. Here we develop exact numerical expression for the outcomes of race model simulations with a single shared threshold and an arbitrary number of accumulators that is based on calculating the distribution of accumulator values across an ensemble of trials.

Consider first the temporal evolution of a single accumulator, distributed on $\boldsymbol{v}$, in the absence of a threshold. For all trials, at time step $\boldsymbol{t}=\mathbf{0}$, the accumulator begins with a value$\boldsymbol{v}=\mathbf{0}$. The probability distribution for this accumulator across an ensemble of trials is therefore defined by the Dirac delta function centered on zero.

$\boldsymbol{P}\left( \boldsymbol{v},\boldsymbol{t}=\mathbf{0} \right)=\boldsymbol{\delta}(\mathbf{0})$ (1)

- At the next time step,$\boldsymbol{t}=\mathbf{1}$, a random value is drawn from a Gaussian distribution that is distributed on $\boldsymbol{v}$ and which has a mean equal to $\boldsymbol{\mu}$ and a standard deviation equal to $\boldsymbol{\sigma}$. This value is added to the accumulator. If $\boldsymbol{G}(\boldsymbol{v},\boldsymbol{\mu},\boldsymbol{\sigma})$ is the function describing this normalized Gaussian distribution, then the distribution of accumulator values at $\boldsymbol{t}=\mathbf{1}$, across an ensemble of trials is given by the convolution (denoted by $*$) of the normalized probability distribution $\boldsymbol{P}(\boldsymbol{v},\boldsymbol{t}=\mathbf{0}$) with the distribution, $\boldsymbol{G}$. Thus:

$\boldsymbol{P}\left( \boldsymbol{v},\boldsymbol{t}=\mathbf{1} \right)=\boldsymbol{P}\left( \boldsymbol{v},\boldsymbol{t}=\mathbf{0} \right)*\boldsymbol{G}\left( \boldsymbol{v},\boldsymbol{\mu},\boldsymbol{\sigma} \right)$ (2)

$\boldsymbol{P}\left( \boldsymbol{v},\boldsymbol{t}=\mathbf{2} \right)=\boldsymbol{P}\left( \boldsymbol{v},\boldsymbol{t}=\mathbf{1} \right)*\boldsymbol{G}\left( \boldsymbol{v},\boldsymbol{\mu},\boldsymbol{\sigma} \right)$ (3)

- And in general, with no threshold, the distribution of accumulator values across an ensemble of trials at an arbitrary time,$\boldsymbol{t}$, is given by the following recurrence relation:
- $\boldsymbol{P}\left( \boldsymbol{v},\boldsymbol{t} \right)=\boldsymbol{P}\left( \boldsymbol{v},\boldsymbol{t}-\mathbf{1} \right)*\boldsymbol{G}\left( \boldsymbol{v},\boldsymbol{\mu},\boldsymbol{\sigma} \right)$ (4)

Including a threshold necessitates that we modify this formulation. Because each trial of a race model ends when the value of the accumulator meets or exceeds the threshold value, $\boldsymbol{T}$, only that subset of trials during which $\boldsymbol{v}<\boldsymbol{T}$ at time $\boldsymbol{t}-\mathbf{1}$ can contribute to an ensemble distribution at time $\boldsymbol{t}$. The influence of a threshold on can be accounted for by truncating $\boldsymbol{P}\left( \boldsymbol{v},\boldsymbol{t}-\mathbf{1} \right)$, removing the portion of the distribution that lies beyond $\boldsymbol{T},$and then renormalizing prior to its convolution with $\boldsymbol{G}.$ Specifically, if $\boldsymbol{U}\left( \boldsymbol{v},\boldsymbol{T} \right)$ is a Heaviside function, $\boldsymbol{U}\left( \boldsymbol{v},\boldsymbol{T} \right)=\left\{ \begin{aligned} \mathbf{0},\boldsymbol{v}<\boldsymbol{T} \\ \mathbf{1},\boldsymbol{v}\geq\boldsymbol{T} \end{aligned} \right.$, then:

$\boldsymbol{P}'\left( \boldsymbol{v},\boldsymbol{t}-\mathbf{1} \right)=\frac{(\mathbf{1}-\boldsymbol{U}(\boldsymbol{v},\boldsymbol{T}))\cdot\boldsymbol{P}\left( \boldsymbol{v},\boldsymbol{t}-\mathbf{1} \right)}{\int_{-\infty}^{\infty} (\mathbf{1}-\boldsymbol{U}(\boldsymbol{v},\boldsymbol{T}))\cdot\boldsymbol{P}\left( \boldsymbol{v},\boldsymbol{t}-\mathbf{1} \right)\boldsymbol{dv}}$ (5)

- $\boldsymbol{P}\left( \boldsymbol{v},\boldsymbol{t} \right)=\boldsymbol{P}'\left( \boldsymbol{v},\boldsymbol{t}-\mathbf{1} \right)*\boldsymbol{G}\left( \boldsymbol{v},\boldsymbol{\mu},\boldsymbol{\sigma} \right)$ (6)

Two particular values will be important for us to compute the distribution of times at which an accumulator reaches the threshold across an ensemble of trials: 1) $\boldsymbol{A}\left( \boldsymbol{t} \right),$the proportion of trials at time $\boldsymbol{t}$ for which the accumulator has a value $\boldsymbol{v}\geq\boldsymbol{T}$; and 2) $\boldsymbol{B}\left( \boldsymbol{t} \right)$, the compliment of $\boldsymbol{A}\left( \boldsymbol{t} \right),$ which reflects the proportion of accumulator values, at time $\boldsymbol{t}$, across an ensemble of trials that are lower than the threshold.

$\boldsymbol{A}\left( \boldsymbol{t} \right)=\int_{\boldsymbol{T}}^{\infty} \boldsymbol{P}(\boldsymbol{v},\boldsymbol{t})\boldsymbol{dv}$ (7)

- $\boldsymbol{B}\left( \boldsymbol{t} \right)=\mathbf{1}-\boldsymbol{A}(\boldsymbol{t})$ (8)

Finally, the probability of the accumulator value exceeding the threshold at time $\boldsymbol{t}$ is equal to the probability that the accumulator value is greater than the threshold at $\boldsymbol{t}$ multiplied by the probability that the accumulator has not had a value greater than the threshold at any time in the past. That is:

- $\boldsymbol{R}\left( \boldsymbol{t} \right)=\boldsymbol{A}\left( \boldsymbol{t} \right)\cdot\prod_{\boldsymbol{t}^{'}=\mathbf{0}}^{\boldsymbol{t}^{'}=\boldsymbol{t}-\mathbf{1}} \boldsymbol{B}\left( \boldsymbol{t}^{'} \right)$ (9)
- We note that this general approach to determining the probability distributions of drift diffusion threshold crossings can be simply altered to account for two boundary conditions as in a typical drift diffusion model.
- An exact expression for the probability distributions of reaction times and errors in a high-dimensional race model framework
- For our purposes we were concerned with determining the distribution of reaction times and errors when a group of accumulators, indexed by $\boldsymbol{i}$, race toward a common threshold. The formulation outlined above may be expanded to account for this situation. Equation (3) can be rewritten as
- $\boldsymbol{P}_{\boldsymbol{i}}\left( \boldsymbol{v},\boldsymbol{t} \right)=\frac{(\mathbf{1}-\boldsymbol{U}(\boldsymbol{v},\boldsymbol{T}))\cdot\boldsymbol{P}_{\boldsymbol{i}}\left( v,\boldsymbol{t}-\mathbf{1} \right)}{\int_{-\infty}^{\infty} (\mathbf{1}-\boldsymbol{U}(\boldsymbol{v},\boldsymbol{T}))\cdot\boldsymbol{P}_{\boldsymbol{i}}\left( \boldsymbol{v},\boldsymbol{t}-\mathbf{1} \right)}*\boldsymbol{G}\left( \boldsymbol{v},\boldsymbol{\mu}_{\boldsymbol{i}},\boldsymbol{\sigma}_{\boldsymbol{i}} \right)$ (10)

The same logic as before dictates that the probability of a particular accumulator $\boldsymbol{i}$ having a value that is greater than or equal to the threshold at time $\boldsymbol{t}$ is given by:

- $\boldsymbol{A}_{\boldsymbol{i}}\left( \boldsymbol{t} \right)=\int_{\boldsymbol{T}}^{\infty} \boldsymbol{P}_{\boldsymbol{i}}(\boldsymbol{v},\boldsymbol{t})\boldsymbol{dv}$ (11)

Similarly, the probability that accumulator $\boldsymbol{i}$ has a value below the threshold is given by:

- $\boldsymbol{B}_{\boldsymbol{i}}\left( \boldsymbol{t} \right)=\mathbf{1}-\boldsymbol{A}_{\boldsymbol{i}}(\boldsymbol{t})$ (12)
- A simulated trial in a race model is terminated when at least one accumulator crosses the threshold. Therefore, we must determine the probability,$\boldsymbol{A}(\boldsymbol{t})$, that one or more accumulators will cross the threshold at time$\boldsymbol{t}$. This can be equivalently interpreted as one minus the probability that none of the accumulators will cross the threshold at time$\boldsymbol{t}$, or:
- $\boldsymbol{A}\left( \boldsymbol{t} \right)=\mathbf{1}-\prod_{\boldsymbol{i}} \boldsymbol{B}_{\boldsymbol{i}}(\boldsymbol{t})$ (13)

The probability,$\boldsymbol{R}\left( \boldsymbol{t} \right)$, of a threshold crossing by any accumulator occurring at time $\boldsymbol{t}$ is equal to the probability that some accumulators have crossed the threshold multiplied by the probability that none of the accumulators have crossed the threshold up to that point. That is:

- $\boldsymbol{R}\left( \boldsymbol{t} \right)=\boldsymbol{A}\left( \boldsymbol{t} \right)\cdot\prod_{\boldsymbol{i}} \prod_{\boldsymbol{t}^{'}=\mathbf{0}}^{\boldsymbol{t}^{'}=\boldsymbol{t}-\mathbf{1}} \boldsymbol{B}_{\boldsymbol{i}}(\boldsymbol{t}^{'})$ (14)

In our simulations, a memory-guided saccade is generated to the location that corresponds to whichever accumulator reaches the threshold value first. The way that the distribution of responses changes across time determines the nature of the speed-accuracy trade-off for a given set of parameters. The distribution of responses at each time point can be determined from $\boldsymbol{A}_{\boldsymbol{i}}\left( \boldsymbol{t} \right)$. Specifically, assuming that there is at least one non-zero value in $\boldsymbol{A}_{\boldsymbol{i}}\left( \boldsymbol{t} \right)$ for a given $\boldsymbol{t}$, the distribution of responses is given by:

$\boldsymbol{M}\left( \boldsymbol{i},\boldsymbol{t} \right)=\frac{\boldsymbol{A}_{\boldsymbol{i}}\left( \boldsymbol{t} \right)}{\sum_{\boldsymbol{i}} \boldsymbol{A}_{\boldsymbol{i}}\left( \boldsymbol{t} \right)}$ (15)

Where $\boldsymbol{M}\left( \boldsymbol{i},\boldsymbol{t} \right)$ is the probability of accumulator $\boldsymbol{i}$ crossing the threshold, thus completing a trial, at time $\boldsymbol{t}$.

Computing the response distributions for ensembles of trials with variable drift rate and response threshold.

Response distributions arising from ensembles of trials with variable threshold and drift rates were determined by taking the weighted sum of response distributions, computed separately across a range of gain and threshold values. For instance, in order to determine the reaction time histogram that would result from the response threshold varying across trials, with a uniform distribution, between 10 and 20, we first computed the histograms resulting from threshold values at the ends of the interval, 10 and 20. If the Pearson’s correlation coefficient between the histograms was less than 0.925 then we would recursively bisected the interval and compute reaction time histograms until all adjacent histograms were correlated with a value greater than 0.925. To illustrate: the first bisection would require computing the reaction time distribution at a threshold of 15. The second bisection would compute RT distributions at threshold values of 12.5 and 17.5. Thus, after the second round, RT distributions would have been computed at evenly spaced threshold values of 10, 12.5, 15, 17.5 and 20. This same procedure was followed for gain (drift rate) variability. The correlation coefficient threshold of 0.925, the value at which recursive bisection was halted, was selected because it provided close agreement between numerically computed and simulated speed-accuracy curves (S1 Fig).
