# Supplementary figures and images for: Working memory improves developmentally as neural processes stabilize
Source: PLoS One. 2019 Mar 7;14(3):e0213010. doi: 10.1371/journal.pone.0213010 (PMC6405198; doi:10.1371/journal.pone.0213010)

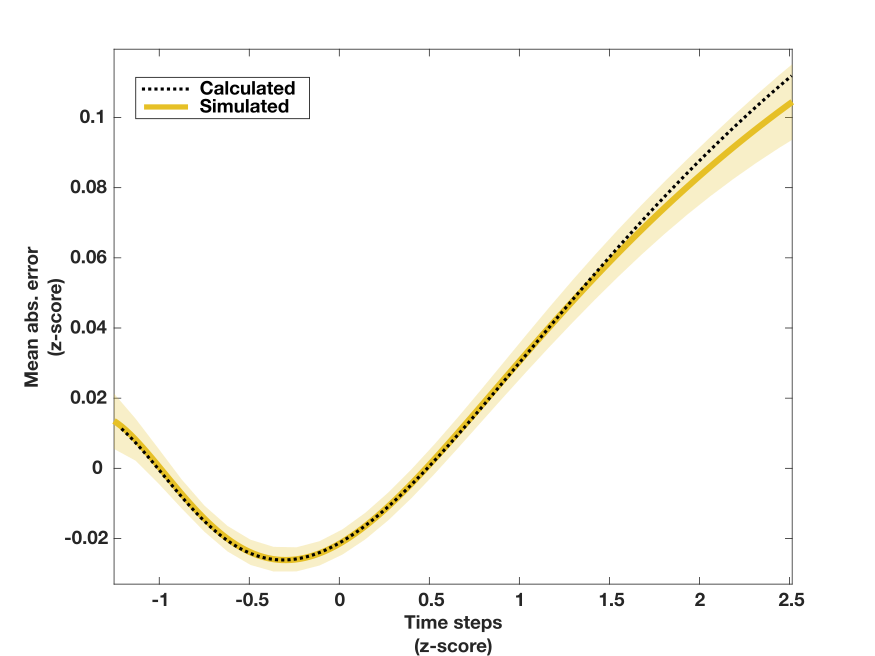

Supplement: S1 Fig — To verify the performance of our numerical calculations, we simulated 6×106 trials using a drift diffusion/race model in which parameters were selected to match those determined to fit the empirical speed accuracy data best. The yellow line depicts the best fitting 15th order polynomial fit to the simulated speed-accuracy curve. The light yellow envelope represents the 95% prediction interval. The dashed line corresponds to the calculated speed-accuracy curve. (TIFF) [file pone.0213010.s002.tiff]
